# Routine Herd Health Data as Cow-Based Risk Factors Associated with Lameness in Pasture-Based, Spring Calving Irish Dairy Cows

**DOI:** 10.3390/ani9050204

**Published:** 2019-04-29

**Authors:** Joris R. Somers, Jon N. Huxley, Michael L. Doherty, Luke E. O’Grady

**Affiliations:** 1 School of Veterinary Medicine, University College Dublin, D04W6F6 Dublin, Ireland; michael.doherty@ucd.ie (M.L.D.); luke.ogrady@ucd.ie (L.E.O.); 2 School of Veterinary Science, Massey University, Palmerston North 4442, New Zealand; j.huxley@massey.ac.nz

**Keywords:** dairy cow, lameness, risk factors, pasture, seasonal

## Abstract

**Simple Summary:**

Dairy cow lameness is considered one of the most important animal welfare and economic concerns for the dairy industry. Cow-based risk factors for lameness are not well described, especially in comparison to herd-level risk factors such as housing environment and roadway condition. This study investigates the use of routinely gathered herd health data as cow-based risk factors for lameness in dairy cows. A total of 1715 cows in 10 pasture-based Irish dairy herds were monitored for lameness during the spring and summer of 2013 and 2014 as part of the University College Dublin herd health programme. Herd health monitoring data analysed to identify potential risk factors for lameness consisted of milk production data, genetic merit information, calving details, peri-parturient disease records and body condition scores. This analysis showed a significant effect of increasing parity, lower body condition score at calving and excessive body condition loss after calving on the risk of cows being diagnosed as lame during the lactation. In conclusion, routinely gathered herd health monitoring data can be used to identify cows at increased risk of being lame and to implement lameness control strategies.

**Abstract:**

Herd-level risk factors related to the cow’s environment have been associated with lameness. Uncomfortable stall surface and inadequate depth of bedding as well as abrasive alley way surface are contributing factors to increased levels of lameness. Access to pasture has been found as having a beneficial effect on cows’ locomotion. However, dairy cattle managed under grazing conditions are exposed to a different set of risk factors for lameness, mainly associated with cow tracks. Cow-based risk factors for lameness are not as clearly defined as the herd level risk factors. The objective of the present study was to use routine herd health monitoring data to identify cow-based risk factors for lameness and utilise this information to indicate cows at risk of developing lameness in the first 150 days of lactation. Lameness data were gathered from 10 pasture-based dairy herds. A total of 1715 cows were monitored, of which 1675 cows were available for analysis. Associations between lameness status and potential cow-level risk factors were determined using multivariable logistic regression. Parity 3 and 4 + cows showed odd ratios (OR’s) for lameness of 3.92 and 8.60 respectively (95% confidence interval (CI) 2.46–6.24; 5.68–13.0). Maximum loss of Body condition score (BCS) after calving exhibits OR’s for lameness of 1.49 (95% CI 1.08–2.04) if cows lost 0.5 in BCS after calving and 2.26 (95% CI 1.30–3.95) for cows losing more than 0.5 BCS. Animals calving in BCS 3.25 and ≥ 3.5 had correlating OR’s of 0.54 (95% CI 0.34–0.87) and 0.33 (95% CI 0.16–0.65) for being lame compared to cows calving with BCS ≤ 2.75. Data gathered as part of herd health monitoring can be used in conjunction with lameness records to identify shortcomings in lameness management. Findings and recommendations on lameness management can be formulated from readily available information on cow-based risk factors for lameness.

## 1. Introduction

Dairy cow lameness is considered one of the most important animal welfare and economic concerns for the dairy industry [1]. Estimates of the percentage of cows that will be affected by lameness during their productive life approach 50% [2]. These estimates do not include subclinical cases that may be overlooked but may still have a negative impact on productivity. Herd-level risk factors related to the cow’s environment have been associated with lameness. Stall surface and depth of bedding as well as alley way surface are often mentioned as contributing factors to increased levels of lameness [3]. The prevalence of lameness in cows housed on deep sand bedded cubicles was lower compared to cows housed on rubber mattress cubicles [4]. Chapinal et al. [5] showed an increased risk for lameness if the proportion of cubicles with faecal contamination increased by 10%. A protective effect on the occurrence of lameness is seen in conjunction with larger herd size, deep bedding used in the stalls, rubber covered floors in the alleys leading to the milking parlour and if cows had access to pasture [5]. Access to pasture has been repeatedly found as having a beneficial effect on cows’ locomotion. In general, cows kept on pasture are less likely to develop locomotion problems [6,7]. Rutherford et al. [8] found lower levels of lameness in herds with longer summer grazing periods. A period of pasture grazing for cows normally cubicle housed showed improvements in locomotion scores, particularly in clinically lame cattle [9,10]. However, dairy cattle managed under grazing conditions are exposed to a different set of risk factors for lameness. The cow track maintenance, the distance travelled along this track and the speed at which cows are forced to move have all been identified as impacting on lameness prevalence in grazing dairy herds [11,12]. Additionally, Ranjbar et al. [13] associated increased average daily rainfall with higher levels of lameness in pasture-based dairy herds. A result attributed to prolonged exposure to moisture making the claw softer and more susceptible to injury [13].

Cow-based risk factors for lameness are not as clearly defined as the herd level risk factors. Untangling the cause-and-effect association is often difficult but it has been shown that high yielding cattle are more prone to develop lameness [14,15]. Cows in low body condition, lighter in live body weight and cows losing excessive body condition are also at a higher risk of becoming lame [14,16,17]. Age of cattle is also mentioned as a risk factor for lameness in a dual capacity. Lower age at first calving is associated with increased locomotion scores, but this may be a reflection of a more intensive production system [8]. Increasing lactation number is the second format in which a cow’s age can affect her susceptibility to lameness [8,10].

The objective of the present study was to use information gathered through routine herd health monitoring to identify cow-based risk factors for lameness previously unreported in pasture-based, seasonally breeding dairy herds and utilise this information to indicate cows at risk of developing lameness in the first 150 days of lactation.

## 2. Materials and Methods 

### 2.1. Study Design

This prospective observational study was carried out on 10 commercial Irish dairy farms during the spring and summer of 2013 and 2014. These 10 farms were part of an on-going herd health management programme conducted by University College Dublin (UCD).

Data recorded as part of the herd health programme were monthly milk recording, including yield to date, predicted 305 day energy corrected milk (ECM) yield, somatic cell count and milk constituents; Economic breeding index (EBI), a single figure profit index aimed at identifying the most genetically profitable bulls and cows for breeding replacements [18]; Calving date, calving difficulty and peri-parturient disease events; Body condition score (BCS) [19] at calving, pre-breeding, services, pregnancy diagnoses and drying off; Ultrasound-based pre-breeding examination, pregnancy diagnosis at 30 and 60 days after insemination and anoestrous examination.

### 2.2. Farm and Animal Selection

The 10 farms involved in this study were located in Co. Kildare and Co. Wicklow, Ireland. The predominant breed on these farms was Holstein-Friesian. All the farms used seasonal breeding, with the breeding season occurring between the middle of April and the middle of August each year. As a result, the calving period on these farms occured between January and June and cows were turned out to grass immediately after calving or as soon as weather conditions and grass growth permitted. Ireland experiences a temperate climate and does not suffer from the extremes of temperature experienced by many other countries of similar latitude [20]. Average maximum temperatures reported by Met Eireann for the time of year the present study was carried out varied between 7.8 °C and 20.1 °C. Average monthly rainfall reported for the same period was between 34.4 mm and 130.2 mm [20]. All farms were visited by a UCD veterinarian every 21 days during the spring calving and breeding season. During these visits the main focus was on the fertility performance and the nutritional management of the cows. Concentrate supplementation in the milking parlour was applied by all 10 farms during times of the grazing season when energy intake from grass alone was not enough to fulfil the cow’s energy requirements for maintenance and production. Concentrates consisted of rolled barley with protein levels ranging from 16% to 20%. The level of concentrates supplemented to the cows’ diet was based on a combination of milk production records and BCS data. Table 1 provides an overview of farm details, including the predicted 305-day ECM yield and lameness assessment results. The predicted 305-day ECM yield is calculated based on adjusted milk fat and milk protein levels of 3.5% and 3.2% respectively.

Only cows calved between 1 January and 30 June, 2013 or 2014 and declared for breeding in the spring breeding herd were included in the study.

### 2.3. Lameness Recording and Management

Lameness data were gathered by means of serial locomotion scoring of all the animals in the breeding herd. Between 25 February 2013 and 1 September 2014, all 10 farms were visited 3 times each year by the same UCD veterinarian. Visits were scheduled in order to facilitate cows being locomotion scored once within 60 days in milk (DIM), once between 60 and 120 DIM and once after 120 DIM. Cows were identified by freeze brand number and locomotion scored by the same observer (JS) for all ten farms as they exited the milking parlour after morning milking using the 5-point scale described by Sprecher et al. [21]. This scale, based on a combination of back posture and gait assessment of cows walking on a level concrete floor, categorises cows with locomotion score (LS) 1 as normal, LS 2 as mildly lame, LS 3 as moderately lame, LS 4 as lame and LS 5 as severely lame. Farmers were given a list of lame cows at the end of each scoring session. Cows on this list were submitted for treatment by the same professional foot trimmer for all 10 farms within 8 days of the scoring session.

### 2.4. Data Management

The data gathered as part of the herd health programme were recorded on the farming software package Herd Master (Irish Farm Computers Ltd, Kells, Co. Meath, Ireland) These data were exported to a Microsoft Office Excel 2010 (Microsoft Corp. Redmond, WA, USA) spreadsheet for calculating the reproductive parameters and evaluating the reproductive performance of the herds at the end of the breeding season. The data obtained during the locomotion scoring visits were added to this spreadsheet.

Animals were categorised into 4 lactation groups representing 1st lactation cows (*n* = 542), 2nd lactation cows (*n* = 387), 3^rd^ lactation cows (*n* = 283) and 4^th^ or greater lactation cows (*n* = 503) respectively. Month of calving was based on the calving date provided by the herd manager, calving dates for April, May and June were combined as the late calving cows. BCS at calving was categorised as ≤ 2.75 (*n* = 223), 3 (*n* = 694), 3.25 (*n* = 613) and ≥ 3.5 (*n* = 146). Maximum BCS loss after calving was grouped as < 0.5 (*n* = 1057), 0.5 (*n* = 500) and > 0.5 (*n* = 118). A case of lameness was defined as an animal with a LS ≥ 3 on the 1–5 scale at least once during the course of the lactation. Each case of lameness was further identified as being observed lame for the first time within 60 DIM, between 60 and 120 DIM and after 120 DIM.

Statistical analysis was carried out in Stata 13.0 (Stata Corp, College Station, TX, USA). Descriptive analyses were performed for all the variables of interest. Associations between lameness status and potential cow-level risk factors were determined using multivariable logistic regression. In a first step, univariable analysis of the association between each hypothesised risk factor and the risk of lameness was performed using logistic regression. Factors with a trend toward significance (*p* < 0.20) were initially considered for inclusion in the multivariable analysis. Secondly, correlations between significant independent variables (*n* = 8) were calculated using the Spearman rank correlation coefficient. When a correlation coefficient > 0.5 between two variables was found, only one was selected for inclusion in the model based on biological plausibility, fewer missing values and reliability of measurement. In this case, peak milk yield was found to be highly correlated with 305-day ECM yield (r = 0.99) and was not retained in the model. EBI for fertility was strongly correlated with overall EBI (r = 0.67) and was omitted from further analysis. The statistical model was built by removing non-significant variables by manual backwards stepwise elimination and confounding was checked for. Confounding would arise where the model is not adjusted for variables related to both the risk factor and the outcome, resulting in false associations. The stepwise elimination process was stopped once all remaining variables were significantly (*p* < 0.05) contributing to the model. Lactation group and 305-day ECM yield were strongly correlated and 2 multivariable logistic regression models were designed retaining either of these two variables. The model likelihood ratio χ^2^ and goodness-of-fit tests for both models were compared and lactation group was chosen over 305-day ECM yield as the variable to be retained in the final model due to a better performance of that model. Two-way interactions between pairs of variables were evaluated using a Wald test to compare models with and without the interaction term included. No significant interaction terms were identified in this way. Eliminated variables were then manually re-entered in the model. If addition or elimination of a variable altered the model estimates by more than 20%, confounding was considered to be present and the variable was retained in the model. No confounders were detected in this way. The variable Farm was forced into the multivariable model to correct for a potential confounding effect. The fit of the multivariable logistic regression model was assessed using the Hosmer–Lemeshow goodness-of-fit test and the Pearson χ^2^ test. Both tests showed an overall good fit of the model.

## 3. Results

### 3.1. Descriptive Results

Table 1 provides an overview of farm details, including the predicted 305-day ECM yield and lameness assessment results. Table 2 shows the study population descriptive statistics. A total of 1715 cows were present in the study population. Eighteen percent of the cows calved in January, 44.1% calved in February, 25.6% calved in March and 12.2% calved in April, May or June. First lactation cows made up 31.6% of the population (*n* = 542). The median number of locomotion scores recorded per cow was 3 and 19.7% of cows were identified as LS ≥ 3 during the study (*n* = 337) and subsequently categorised as lame. Forty cows, randomly present on all ten farms, were excluded from the study due to missing BCS data. Consequently 1675 cows were available for analysis.

### 3.2. Multivariable Logistic Regression Results

Of all the risk factors presented, month of calving group was the only variable not showing a significant association with lameness status in the univariate analysis (*p* = 0.89) and was subsequently not used in further multivariable analysis. After accounting for the correlations described earlier, the final model consisted of the variables Farm, Lactation group, BCS at calving group and Maximum BCS loss after calving group (Table 3). Lactation group showed the largest odds ratios (ORs), parity 2 cows showed a near significant OR and parity 3 and 4+ cows showed a significant OR of 1.52, 3.92 and 8.60 respectively (95% confidence interval (CI) 0.92–2.50; 2.46–6.24; 5.68–13.0 respectively). The OR for maximum loss of BCS after calving group was 1.49 (95% CI 1.08–2.04) if cows lost 0.5 in BCS after calving and 2.26 (95% CI 1.30–3.95) for cows losing more than 0.5 BCS after calving. Increased BCS at calving group showed a protective association with lameness status. Animals calving in BCS 3.25 and ≥ 3.5 respectfully had an OR of 0.54 (95% CI 0.34–0.87) and 0.33 (95% CI 0.16–0.65) of being lame compared to cows calving with BCS ≤ 2.75. The BCS 3 at calving group did not show a significant OR (0.82; CI 0.56–1.20) but the result was in line with the decreasing OR for lameness development identified for increasing BCS at calving.

## 4. Discussion

The typical Irish dairy herd is managed as a pasture-based seasonal system, reflected by the vast majority of cows in this study having calved in the months January through March (87.8%) and 12.2% of cows calved in April, May or June. The point prevalence of lameness during the Irish grazing season was previously reported at a level of 11.6% [22]. A total of 337 cows (19.7%) were identified with a case of lameness during the study. The median DIM cows were first identified as a case of lameness (84.4 days; SD: 53.2 days) is similar to data reported in studies from England and Canada [23,24]. Chesterton et al. [11] described risk factors for lameness in dairy cattle under grass-based production systems in New Zealand. Breed was the only cow-based risk factor identified by these authors [11]. Holstein-Friesian was the predominant breed in all the herds included in the current study preventing evaluation of that risk factor in our data. Farm was included in the final multivariable logistic model to account for farm effects such as feeding and lameness management. Lameness management was different between the 10 study farms [25] and can often be used as an overall indication of animal husbandry management on the farm. Nonetheless, Farm showed a near significant association with lameness for farms 5 and 8 and a significant association for farm 10 when comparing to farm 1 as the reference in the final model. Farm 1 was chosen as the reference factor because it had the lowest proportion of lame cows (12.5%). Lactation group showed the largest associations with lameness of all the variables in the model. This indicates that increasing parity increases the susceptibility of cows to lameness and confirms the finding by Solano et al. [23] that cows in their third or greater lactation had three times the odd of being lame compared to first lactation cows. This effect was also reported in another study, but the relationship began to level-off after lactation 6 [26]. It was suggested by Hirst et al., [26] that cows surviving to later lactations were less prone to lameness and hence earlier culling. By grouping cows in parity 4 and older, that distinction could not be made in the current study. However, Pötzsch et al. [27] were unable to detect a significant difference in the LS of animals in lactation 2 to 5. Maximum BCS loss after calving was associated with increased risk of lameness in the current study. BCS loss is a reasonably accurate measure of negative energy balance and nutritional management of the cow and has been associated with health disorders such as lameness [28]. In the present study, cows losing 0.5 in BCS after calving were 1.5 times more likely to be identified lame and cows losing more than 0.5 in BCS after calving were 2.3 times more likely to be lame. In a study investigating the effect of milk yield and live weight loss on the risk of lameness in a seasonally calving, pasture fed dairy herd in New Zealand, an additive interaction between live weight loss and milk yield was found. But the risk difference remained the highest for live weight loss across low and high yielding cows [14]. Those authors estimated a reduction in lameness incidence risk by approximately 3% if weight loss was prevented, representing 23% of the lameness incidence risk in the study herd as being attributable to exposure to live weight loss. The present study was unable to untangle to temporal association between BCS loss after calving and lameness as it was not clear from the gathered data when the BCS loss occurred in relation to the lameness event. However, a recent study reported that the loss of BCS increased a cow’s probability of subsequently becoming lame and decreased her likelihood of recovery over the following 15 days as the key finding [17]. Lim et al. [17] also reported that the extent of the loss of BCS was reflected in the probability of transition from non-lame to lame. In addition to loss of BCS having a detrimental effect on the occurrence and recovery from lameness, the mentioned study also supported earlier studies [16,23] in concluding that low BCS per se is a risk for lameness. The current study was able to show a similar association between BCS at calving and lameness. Cows in a greater BCS at calving were at a lower risk of being identified lame during the subsequent lactation.

The Irish dairy industry is focused on milk production on a grass-based diet and as a result the findings of this study may not apply to other herd management systems. Furthermore, the data analysed were collected over the course of two years in a small number of herds. However, as access to pasture has been associated with aiding lame cows to recover and reducing lameness prevalence [5,9], the present study has its value in establishing the association between cow-based risk factors for lameness under grass-based dairy production systems. Given the consistency between our results and those reported in the literature, we consider that cow-based risk factors for lameness are generalisable across dairy production systems. Further research, incorporating a larger sample of pasture-based Irish dairy herds, could facilitate the identification of a wider range of cow-based risk factors that can be recorded as part of herd health monitoring in an effort to reduce the cows’ susceptibility to develop lameness.

## 5. Conclusions

Despite access to pasture having a protective effect on the occurrence of lameness [5], we identified nearly 20% of cows lame during the study. Increasing parity and BCS loss after calving were associated with an increased risk for lameness whereas increased BCS at calving was associated with reduced risk for lameness. The results of this study show that data gathered as part of a routine herd health programme show the potential to be used in conjunction with lameness records to identify shortcomings in the on-farm lameness management. Findings and recommendations on lameness management of different age groups and cows in different nutritional status can be formulated from readily available information on cow-based risk factors for lameness. Further analyses of lameness data and identification of associated risk factors is required to further understand the dynamics of lameness in pasture-based, spring calving dairy herds and to formulate strategies to reduce the incidence of this debilitating condition.

## Figures and Tables

**Table 1:** Overview of the 10 farms. N shows the number of cows from each farm included in the study, mean yield is calculated based on the predicted 305 day energy corrected milk (ECM) yield expressed in kilogram (Kg). Proportion lame is the proportion of cows in the herd identified as lame during the study, the days in milk (DIM) category 1^st^ lame shows the proportion of lame cows identified during each of the 3 DIM categories.

Farm	N	Mean Yield (Kg)	Proportion Lame (%)	DIM Category 1^st^ Lame (%)		
				<60	60–120	>120
1	160	6782.8	12.5	3.7	4.4	4.4
2	302	6184.3	14.2	5.6	6.9	1.7
3	143	6609.4	16.8	4.9	4.9	7.0
4	136	7828.6	19.1	11.0	5.2	2.9
5	200	5875.6	20.5	9.5	4.0	7.0
6	160	6437.3	20.6	7.5	8.1	5.0
7	105	6822.8	21.9	5.7	14.3	1.9
8	242	5998.4	24.8	9.5	7.4	7.9
9	44	5554.9	25.0	9.1	13.6	2.3
10	223	6250.7	25.1	8.1	12.5	4.5

**Table 2:** Study population descriptive statistics of parity, calving date, milk production body condition score (BCS) at calving and BCS loss post calving, economic breeding index (EBI), the number of locomotion scores (LS) recorded per cow during each lactation and days in milk (DIM) cows were identified as lame.

Variable	N (cows)	Mean (SD)	Median	Min/Max ^1^
Lactation number	1715	2.73 (1.74)	2	1/11
1st Lactation	542			
2nd Lactation	387			
3rd Lactation	283			
4th + Lactation	503			
Calving month				
January	309			
February	757			
March	439			
April, May or June	210			
BCS at calving				
≤2.75	223			
3	694			
3.25	613			
≥3.5	146			
Max ^1^ BCS loss post calving				
<0.5	1057			
0.5	500			
>0.5	118			
305day ECM yield (Kg)		6423.9 (1331.1)	6374.5	2792/10242
Peak milk yield (Kg)		33.9 (7.1)	33.7	14.70/53.90
EBI overall (€)		128.0 (42.6)	132.3	-80.9/283.7
EBI fertility (€)		64.3 (32.1)	66.4	-53.8/154.4
No. of LS recorded		2.9 (0.90)	3	1/4
DIM identified lame (days)	337	84.4 (53.2)	73	2/231

^1^ Min = minimum; Max = maximum.

**Table 3:** Summary of the final model of routine herd health data as cow-based risk factors associated with lameness in dairy cows. Lameness was defined as a LS ≥ 3 being awarded during lactation.

Variable	OR^2^	SE	*p* Value	95% CI for ^2^
Intercept	0.06	0.02	<0.0001	0.03 – 0.13
Farm				
1	1	reference		
2	0.97	0.30	0.93	0.53–1.79
3	1.07	0.38	0.85	0.53–2.14
4	1.53	0.54	0.23	0.77–3.05
5	1.63	0.52	0.13	0.87–3.04
6	1.02	0.35	0.96	0.52–1.98
7	1.35	0.51	0.43	0.64–2.85
8	1.77	0.56	0.07	0.96–3.28
9	1.57	0.73	0.33	0.63–3.93
10	1.96	0.60	0.03	1.07–3.59
Lactation group				
Lactation 1	1	reference		
Lactation 2	1.52	0.39	0.10	0.92–2.50
Lactation 3	3.92	0.93	<0.0001	2.46–6.24
Lactation 4+	8.60	1.82	<0.0001	5.68–13.0
BCS at calving group				
≤2.75	1	reference		
3	0.82	0.16	0.31	0.56–1.20
3.25	0.54	0.13	0.01	0.34–0.87
≥3.5	0.33	0.11	0.001	0.16–0.65
Max BCS loss				
<0.5	1	reference		
0.5	1.49	0.24	0.01	1.08–2.05
>0.5	2.26	0.64	0.004	1.30–3.95

^2^ OR = odds ratio; CI = confidence interval.
